# Treatment patterns for advanced therapies in Canadians with moderate-to-severe inflammatory bowel disease: a retrospective cohort analysis

**DOI:** 10.1093/jcag/gwae040

**Published:** 2024-10-29

**Authors:** Laura Targownik, Waqqas Afif, Sunny Singh, Jesse Siffledeen, Christopher Ma, Kevin McHugh, Julie Charbonneau, Louis-Charles Rioux

**Affiliations:** Department of Gastroenterology & Hepatology, University of Toronto, Mount Sinai Hospital, 600 University avenue, Toronto, ON M5G 1X5, Canada; Department of Medicine, McGill University, Montreal General Hospital, Montreal, Quebec H3G 1A4; Division of Gastroenterology, Department of Medicine, University of British Columbia, Kelowna General Hospital, Kelowna, British Columbia; Division of Gastroenterology, University of Alberta, Edmonton, Alberta; Division of Gastroenterology & Hepatology, Department of Medicine, Cumming School of Medicine, University of Calgary, Calgary, Alberta; Division of Gastroenterology & Hepatology, Department of Community Health Sciences, Cumming School of Medicine, University of Calgary, Calgary, Alberta T2N 4N1; AbbVie Corp., Saint-Laurent, Quebec; AbbVie Corp., Saint-Laurent, Quebec; Division of Gastroenterology, University of Montréal, Hôpital Maisonneuve-Rosemont, Montreal, Quebec

**Keywords:** biologics, sequencing, therapeutics

## Abstract

Many patients with inflammatory bowel disease (IBD) show an inadequate response or experience a loss of response to advanced therapies. Guidelines recommend dose optimization and switching among therapies until an optimal treatment response is attained. With several advanced treatments available, we aimed to evaluate the persistence of different therapeutic sequences in IBD.

The RECORDED study was a retrospective cohort study of Canadians with moderate-to-severely active ulcerative colitis (UC) or Crohn’s disease (CD) who had been exposed to more than 1 advanced therapy between May 2015 and April 2021 for UC, and May 2016 and April 2021 for CD. The primary endpoint was time to permanent discontinuation of the first advanced treatment.

Overall, 330 patients had CD and 344 had UC. The most common first-line treatments for CD and UC were adalimumab and infliximab, respectively. The median (95% CI) time to permanent discontinuation of first-line treatment was 12.3 (10.9, 13.6) months in patients with CD and 9.2 (8.2, 10.8) months for those with UC. The most common reason for treatment change across both diseases was lack of efficacy. First-line advanced treatments were optimized in 191 (58.1%) CD patients and 202 (59.1%) UC patients prior to permanent discontinuation. Second-line therapy was typically from a different class compared with the first-line treatment choice.

The RECORDED study provides insights into the real-world sequencing and optimization patterns of advanced treatments in patients with moderate-to-severe IBD in Canada. Lack of efficacy was the most cited reason for switching to a different therapy.

## Introduction

The treatment goal for patients with moderate-to-severely active inflammatory bowel disease (IBD) is to improve clinical symptoms and endoscopic evidence of inflammation, ideally in the form of achieving mucosal healing.^1–3^ Advanced therapies including biologic agents and other targeted immunosuppressant medications are superior to placebo in achieving symptom improvement and endoscopic healing in persons with Crohn’s disease (CD) and ulcerative colitis (UC).^4–12^ A “treat-to-target” approach, where objective assessments of inflammatory activity guide decisions on advanced therapies, is associated with improved rates of symptomatic remission and normalization of biochemical and endoscopic indicators of inflammation, which in turn are associated with a reduced risk of hospitalization and surgery among patients with IBD, compared to making treatment decisions based on symptoms alone.^13–15^

Despite this, the extent of treatment optimization remains poorly characterized in Canada for patients with moderate-to-severely active IBD. Furthermore, there is uncertainty regarding the optimal treatment sequencing of advanced agents, especially having multiple therapeutic options targeting different mechanisms of action. The RECORDED study aims to elucidate modern treatment patterns, focusing on Canadians with Crohn’s Disease and ulcerative colitis exposed to multiple advanced therapies, with a specific interest in drug survival, sequencing, and optimization patterns of advanced treatments.

## Methods

### Study design and setting

The RECORDED study was a retrospective cohort study of IBD patients using advanced therapies whose care was provided at one of 22 Canadian academic, community, and rural treatment centers. The study was conducted in compliance with the guidelines for Good Pharmacoepidemiology Practices in noninterventional studies. Research ethics board approval was obtained at all sites. As this was an analysis of de-identified patient data, informed consent was not required.

### Participants

We included data from adults treated for moderate-to-severely active IBD (as defined by the treating physician) with more than 1 advanced therapy (adalimumab, infliximab, vedolizumab, ustekinumab, and tofacitinib) for at least 1 year (combined), with the first such therapy initiated between May 2015 and April 2021 for UC and between May 2016 and April 2021 for CD.

This timeframe reflected an era when management guidelines recommended a treat-to-target approach for patients with IBD requiring advanced therapies (and covered a period from approval of the first nontumor necrosis factor (TNF) antagonist biologic in 2015, ranging to approval of tofacitinib in Canada for the treatment of UC on October 9, 2018). Dates for Health Canada Notice of Compliance are provided in Supplementary information Table S1. Patients failing at least 1 advanced therapy were included, ensuring precision for secondary analyses and assessing the proportion of patients undergoing treatment optimization before discontinuation. Exclusion criteria comprised prior IBD-related surgery before initiating advanced therapy.

### Variables

Data collected included demographics, disease history and baseline characteristics, advanced therapies used, and any use of IBD-related concomitant medications. Data were also collected regarding the prescribing physician (type of practice, speciality, and years in practice) and healthcare resource utilization (number of clinic visits related to IBD management).

The primary endpoint was time to permanent discontinuation of the first advanced treatment. Secondary endpoints included time to permanent discontinuation of second advanced treatment; time to first treatment modification of first and second advanced treatment and proportion of patients with optimized use of first advanced therapy prior to permanent discontinuation.

The full analysis set (FAS) included subjects who met all the inclusion criteria study and had been treated for IBD (with moderate-to-severe CD or UC) with approved advanced therapies comprising biologics (adalimumab, infliximab, vedolizumab, and ustekinumab) and tofacitinib for at least 1 year.

### Data sources

Data were extracted from eligible patients’ medical records. To ensure similar numbers of patients with UC and CD and reasonable geographic representation, site, and study-specific targets were utilized for the number of CD/UC patients and for each first-line therapy. Treatment centres were instructed to identify eligible patients based on sequential attendance at the clinic, starting with those seen most recently, to ensure a representative sample of their patient pool was identified.

### Study size

The sample size was determined based on the estimated precision and resource limitation. A median time to permanent treatment discontinuation of 36 months was assumed, with no censor (since all subjects will have been treated with 2 biologics per the inclusion criteria) and exponential distribution of the time to treatment discontinuation. Under these assumptions, a sample size of 800 subjects gave a 95% CI with a lower limit of 33.7 months and an upper limit of 38.7 months for a median time of 36 months. As subjects were separated into those with CD and those with UC, with approximately 350 subjects in each cohort, 350 events produced a two-sided 95% confidence interval with a width equal to 7.583 when the estimate of the median survival time is 36 months. This gives a confidence interval with a lower limit of 32.507 and an upper limit of 40.091 months for the median survival time.

### Quantitative variables

Study variables were summarized using descriptive statistics. In addition, age at diagnosis was grouped as less than 18 years, 18-40 years, and over 40 years of age. Implementation of dose optimization was defined by the treating physician per the patient’s medical records. Given the variability in optimization strategies available (eg, change in dose, frequency, or even addition of an immunomodulator) This was recorded as a categorical yes/no rather than pursuing the details of the optimization.

### Statistical methods

The primary endpoint (and similar secondary endpoints) was estimated using the Kaplan–Meier method, including associated two-sided 95% confidence interval and the permanent discontinuation rate at 1 and 2 years. Treatments that were ongoing at the time of data extraction were censored at the date of the chart review. Missing data were not imputed, except for the biologic treatment start/stop date. The start/stop date with only the year was set as missing and excluded from analyses. The start/stop date with a missing day was imputed to the first day of the month. Other endpoints were summarized using descriptive statistics. Associations between patient demographics, disease history, nature of treatment change or physician characteristics, and first- or second-line advanced therapy choice were examined descriptively. Factors associated with discontinuation of second-line therapy were elucidated first by identifying those that were statistically significantly different between patients who remained on their second-line therapy vs those who transitioned to third-line treatment using chi-squared analyses, then performing a logistic regression analysis with those parameters with a positive association based on the results of the Chi-squared analyses.

## Results

### Patient demographics and baseline characteristics

Data were extracted from the medical records of 724 patients from 22 treatment centres, of which 674 (93.1%) were included in the FAS. A list of participating sites may be found in Table S2. Reasons for the exclusion of 50 patient records from the FAS included not meeting at least 1 of the eligibility criteria and incomplete start/stop dates for advanced therapies such that treatment duration could not be determined. Overall, 330 (49.0%) patients had CD and 344 (51.0%) had UC. Mean (SD) age was 42.7 (16.2) years for those with CD and 42.6 (16.6) years for those with UC. Mean (SD) time from diagnosis to initiation of the first advanced therapy was 644 (1409) days for patients with CD and 978 (1943) days for those with UC (Table 1). Mean [SD] time from diagnosis to initiation of first advanced therapy was significantly longer for those patients who recorded 3 advanced therapies than for those who recorded 2 (922 [1897] vs. 753 [1560] days. *P* = .044).

**Table 1.** Patient demographics and baseline characteristics.

	Crohn’s disease (*N* = 330)	Ulcerative colitis (*N* = 344)
*Age, years*		
*n*	330	344
Mean (SD)	42.7 (16.2)	42.6 (16.6)
*Female sex,*		
*n*/*N* (%)	184/330 (55.8)	163/344 (47.4)
*Height, cm*		
*n*	95	116
Mean (SD)	168.9 (10.2)	170.0 (8.9)
*Weight, kg*		
*n*	206	213
Mean (SD)	75.9 (19.7)	74.0 (17.9)
*BMI, kg/m* ^*2*^		
*n*	70	90
Mean (SD)	26.0 (6.9)	25.0 (5.8)
*Age at IBD diagnosis, years*		
*n*	330	343
Mean (SD)	37.9 (16.6)	37.5 (16.9)
*Age group at IBD diagnosis, (n [%])*		
<18 years	24 (7.3)	23 (6.7)
18–40 years	169 (51.2)	183 (53.2)
>40 years	137 (41.5)	138 (40.1)
*Days from IBD diagnosis to initiation of first advanced therapy*		
*n*	175	154
Mean (SD)	644 (1409)	978 (1943)
*Corticosteroid use*		
*n*/*N* (%)	123/330 (37.3)	178/344 (51.7)
*Immunosuppressant use*		
*n*/*N* (%)	83/330 (25.2)	65/344 (18.9)
*Harvey-Bradshaw score*		
*n*	96	NA
Mean (SD)	8.4 (3.77)	
*Total Mayo score*		
*N*	NA	80
Mean (SD)		7.38 (3.43)

Abbreviations: BMI, body mass index; IBD, inflammatory bowel disease; NA, not applicable.

### Physician demographics and baseline characteristics

Physicians at 22 sites participated in the study, 11 of which self-identified as academic, 12 as community and 2 as rural treatment centres (sites could choose more than 1 descriptor). Seven sites were located in the West, 6 in Ontario, 4 in Quebec and 4 in the Atlantic region. The physicians had been in practice for a median (range) of 15 (4-45) years (Table S2). There was a statistically significant correlation between years in practice and type of practice for both CD and UC (*P* < .0001 for both comparisons).

### Advanced therapies by line of treatment

**Figure 1.**
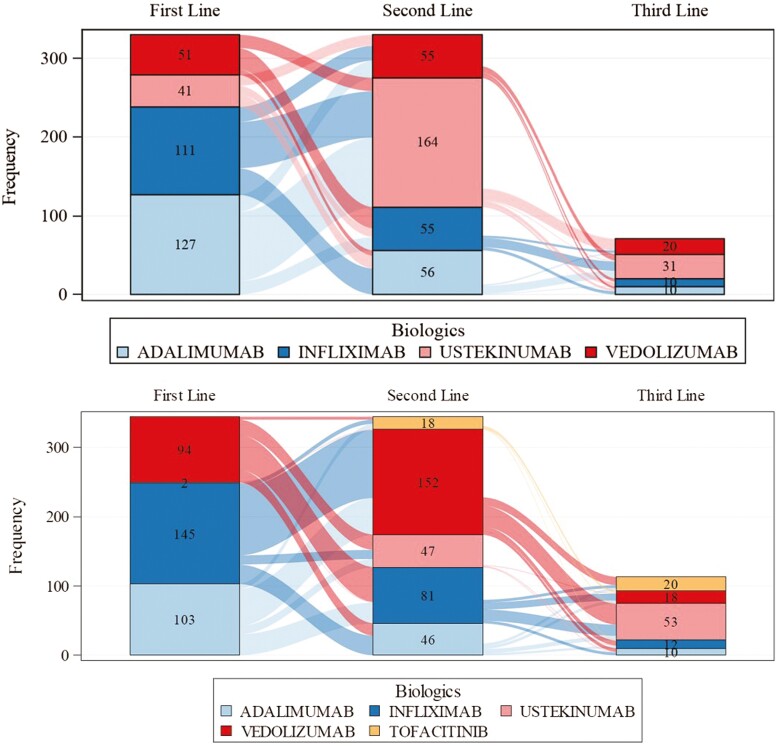
Sankey diagrams depicting associations between first-, second- and third-line treatment choices for patients with Crohn’s disease (upper figure) and ulcerative colitis (lower figure).

For patients with CD, adalimumab was the most commonly used first-line treatment (*N* = 127 [38.5%]), followed by infliximab (*N* = 111 [33.6%]), vedolizumab (*N* = 51 [15.5%]), and ustekinumab (*N* = 41 [12.4%]) (Table 2). For those with UC, more patients received infliximab first line (*N* = 145 [42.2%]) than adalimumab (*N* = 103 [29.9%]), vedolizumab (*N* = 94 [27.3%]), or ustekinumab (*N* = 2 [0.6%]). Due to its recent availability and restrictive reimbursement, tofacitinib was only captured as a second- or third-line treatment in UC. Crohn’s disease patients who failed first-line TNF antagonist therapy were most frequently switched out of class to ustekinumab (94/149 [68%] from adalimumab and 64/120 [53%] from infliximab). Those patients failing ustekinumab first line most frequently switched to either adalimumab (16/41 [39%] of cases) or vedolizumab (14 [34%] of cases). Those failing vedolizumab first line most frequently switched to infliximab (27/52 [52%] of cases) (Figure 1).

**Table 2.** Advanced therapies used by line of treatment.

Treatment (*n*)	1st line	2nd line	3rd line
*Crohn’s disease*	*N* *=* *330*	*N* = *330*	*N* = *71*
Adalimumab	127 (38.5%)	56 (17.0%)	10 (14.1%)
Infliximab	111 (33.6%)	55 (16.7%)	10 (14.1%)
Vedolizumab	51 (15.5%)	55 (16.7%)	20 (28.2%)
Ustekinumab	41 (12.4%)	164 (49.7%)	31 (43.7%)
*Ulcerative colitis*	*N* = *344*	*N* = *344*	*N* = *113*
Adalimumab	103 (29.9%)	46 (13.4%)	10 (8.8%)
Infliximab	145 (42.2%)	81 (23.5%)	12 (10.6%)
Vedolizumab	94 (27.3%)	152 (44.2%)	18 (15.9%)
Tofacitinib	0	18 (5.2%)	20 (17.7%)
Ustekinumab	2 (0.6%)	47 (12.2%)	53 (46.9%)

Tofacitinib was only included if it was prescribed as second-line therapy.

Ulcerative colitis patients who failed first-line TNF antagonists were most frequently switched to vedolizumab (56/107 [52%] from first-line adalimumab and 102/121 [66%] from first-line infliximab). Those failing vedolizumab first line were most frequently switched to infliximab (60/105 [57%]).

### Time to permanent discontinuation of first- and second-line advanced therapy

**Figure 2.**
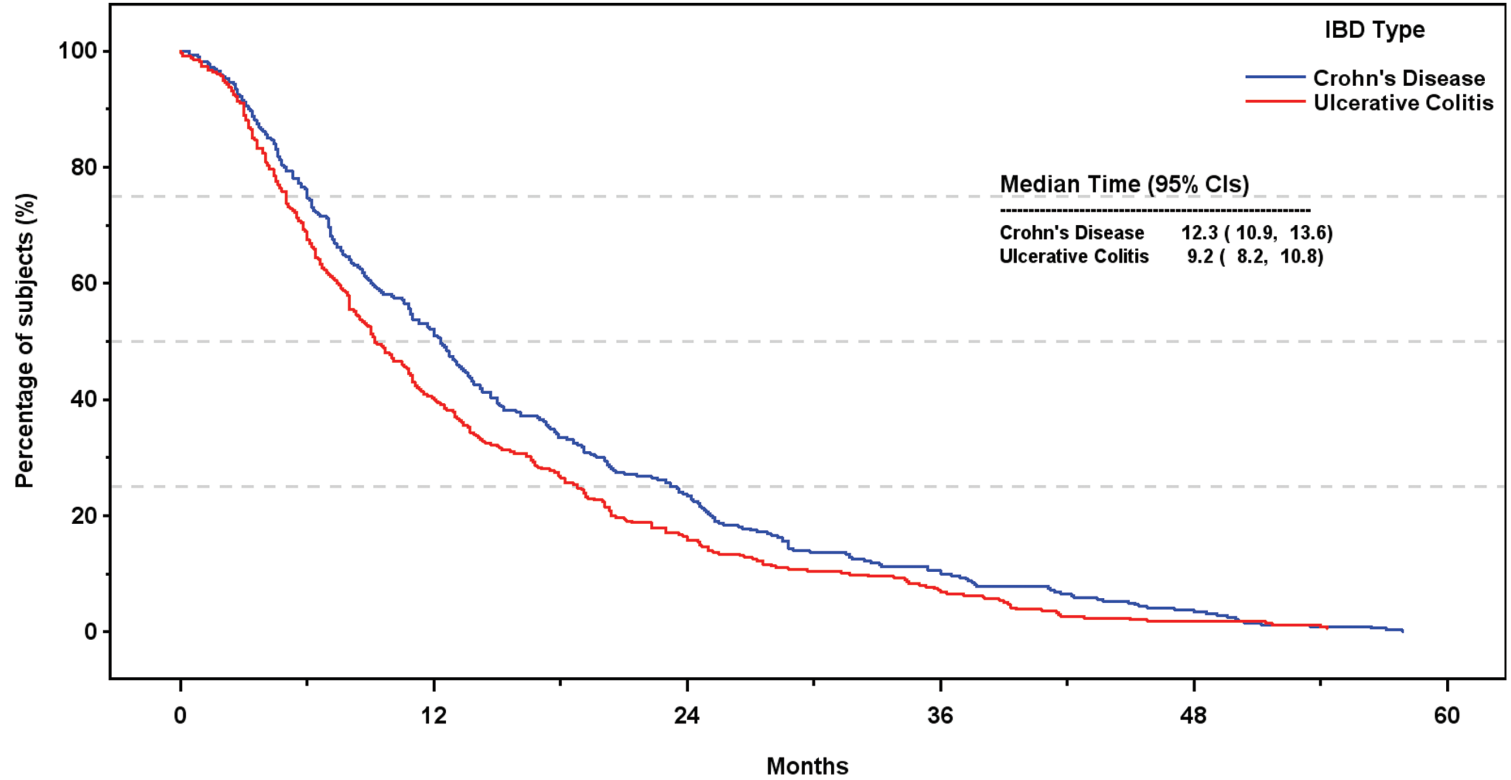
Kaplan–Meier plots of time to permanent discontinuation of first line therapy for patients with Crohn’s disease and ulcerative colitis.

In this cohort of patients who experienced a primary response but then by definition discontinued first-line therapy, the median (95% CI) time to permanent discontinuation of first-line treatment was 12.3 (10.9, 13.6) months in patients with CD and 9.2 (8.2, 10.8) months for those with UC (Figure 2 and Table 3). The median time to permanent discontinuation of first-line treatment was similar for all advanced therapies in patients with CD but more variable for those with UC, ranging from 8.0 (6.8, 10.5) months for adalimumab to 11.5 (9.7, 13.7) months for vedolizumab (Supplementary information Figures S1 and S2; *P* = .909). Patient numbers for ustekinumab in UC were too low to allow meaningful analysis or interpretation.

**Table 3.** Time to permanent discontinuation of first- and second-line advanced therapy.

	Estimated time (months)	Patients
*Crohn’s disease—first advanced therapy*		*N* = *320*
Median (95% CI) time to discontinuation	12.3 (10.9, 13.6)	
Proportion of patients who discontinued by:		
1 year		49.1%
2 years		76.6%
3 years		90.0%
*Crohn’s disease—second advanced therapy*		*N* = *330*
Median (95% CI) time to discontinuation	Not estimable	
Proportion of patients who discontinued by:		
1 year		17.3%
2 years		30.9%
3 years		33.7%
*Ulcerative colitis—first advanced therapy*		*N* = *355*
Median (95% CI) time to discontinuation	9.2 (8.2, 10.8)	
Proportion of patients who discontinued by:		
1 year		60.0%
2 years		84.2%
3 years		93.1%
*Ulcerative colitis—second advanced therapy*		*N* = *342*
Median (95% CI) time to discontinuation	46.7 (34.4, NA)	
Proportion of patients who discontinued by:		
1 year		27.0%
2 years		39.3%
3 years		45.5%

Median time and 95% CIs, and treatment discontinuation rate, are estimated from the Kaplan–Meier analysis. If the treatment was ongoing at the time of chart review, the stop date was right censored at the chart review date.

**Figure 3.**
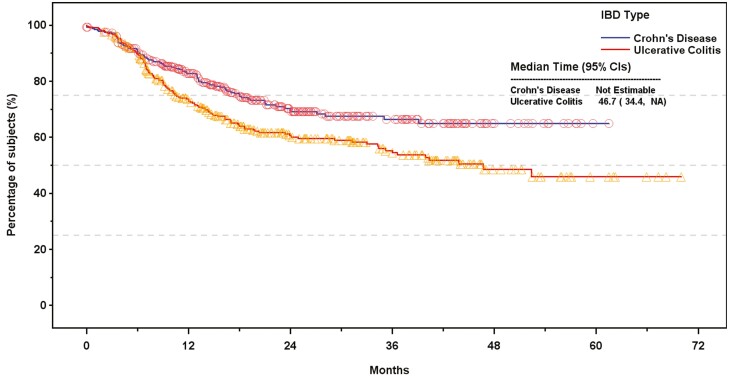
Kaplan–Meier plots of time to permanent discontinuation of second line therapy for patients with Crohn’s disease and ulcerative colitis.

In contrast to first-line therapy (which all patients had to have discontinued), patients who initiated second-line therapy included those who found that treatment effective and tolerable (ie, responders), as well as those who did not (ie, non-responders). This was reflected in the proportion of patients who progressed to the second line (CD: *n* = 330, 100%; UC: *n* = 344, 100%) compared with third-line therapy (CD: *n* = 71, 21.5%; UC: *n* = 113, 32.8%). As a result, median time to permanent discontinuation of second-line treatment was not estimable for CD patients; however, 17.3% of patients with CD discontinued second-line treatment within a year of its initiation, rising to 33.7% by the end of the third year (Table 3). For those with UC, the median (95% CI) time to permanent discontinuation of second-line therapy was 46.7 (34.4, not estimable) months (Figure 3); 27.0% discontinued second-line treatment within a year; 45.5% discontinued within 3 years (Table 3).

### Treatment optimization

First-line advanced treatments were optimized in 191 (58.1%) CD patients and 202 (59.1%) UC patients prior to permanent discontinuation (Table 4). Vedolizumab had the lowest rate of dose optimization in the CD cohort, while the rates of dose optimization were similar for adalimumab, infliximab and vedolizumab in the UC cohort. The proportion of patients who had their treatment optimized prior to its discontinuation ranged from 88.2% for patients with UC receiving ustekinumab to 48.6% for those with CD receiving vedolizumab. Median (95% CI) time to first-line treatment optimization in patients with CD ranged from 5.2 (4.8, 19.9) months for those receiving vedolizumab to 9.0 (5.8, 11.0) months for those receiving adalimumab (Supplementary information Figure S3). For patients with UC, the median (95% CI) time to first-line treatment optimization ranged from 4.1 (2.9, 6.6) months for adalimumab to 6.9 (6.0, 8.5) months for vedolizumab, with infliximab (the most used first-line treatment) intermediate at 5.4 (4.1, 6.5) months (Supplementary information Figure S4).

**Table 4.** Proportion of patients who underwent dose optimization of advanced therapies prior to permanent discontinuation, by line of therapy.

Line of treatment	Dose optimized (*n*/*N* [%])
*Crohn’s disease*	
First	191/329 (58.1)
Second	142/329 (43.2)
Third	27/71 (38.0)
*Ulcerative colitis*	
First	202/342 (59.1)
Second	171/344 (49.7)
Third	59/112 (52.7)

Patients with no mention in their chart of whether (or not) there was a dose or frequency change were excluded from the analysis.

### Reasons for permanent treatment discontinuation of first line advanced therapy

The most common reason for permanent treatment discontinuation of first-line advanced therapy across both cohorts was lack of efficacy (CD: 270/435 [62.1%] events; UC: 400/527 [75.9%] events), followed by adverse events (CD: 102/435 [23.4%] events; UC: 69/527 [13.1%] events) (Table 5). Of note, anti-drug antibodies (included in the “other” category) were cited as the reason for treatment discontinuation in 38 (9.3%) patients receiving infliximab (19 with CD, 19 with UC), 10 (2.8%) patients receiving adalimumab (6 with CD, 4 with UC) and 2 (0.5%) patients receiving vedolizumab (1 with CD, 1 with UC). A further 3 patients receiving infliximab and 2 patients receiving adalimumab had their treatment discontinued due to the inability to achieve therapeutic drug levels. The median time between discontinuation of the first line and initiation of the second line therapy was approximately 3 weeks for both patient cohorts and did not appear to include a wash-out period (Supplementary information Table S3).

**Table 5.** Primary reason(s) for permanent discontinuation by line of therapy.

Primary reason for permanent discontinuation	Crohn’s disease *N* (%)	Ulcerative colitis *N* (%)
*Overall*		
Total events	435	527
Adverse event(s)	102 (23.4)	69 (13.1)
Lack of efficacy	270 (62.1)	400 (75.9)
Other	49 (11.3)	48 (9.21)
Patient request	12 (2.8)	3 (0.6)
Unknown	2 (0.5)	7 (1.3)
*First line*		
Total events	330	344
Adverse event(s)	78 (23.6)	54 (15.7)
Lack of efficacy	205 (62.1)	255 (74.1)
Other	37 (11.2)	30 (8.7)
Patient request	9 (2.7)	2 (0.6)
Unknown	1 (0.3)	3 (0.9)
*Second line*		
Total events	86	135
Adverse event(s)	21 (24.4)	12 (8.9)
Lack of efficacy	54 (62.8)	104 (77.0)
Other	9 (10.5)	16 (11.9)
Patient request	1 (1.2)	1 (0.7)
Unknown	1 (1.2)	2 (1.5)
*Third line*		
Total events	19	42
Adverse event(s)	3 (15.8)	3 (7.1)
Lack of efficacy	11 (57.9)	35 (83.3)
Other	3 (15.8)	2 (4.8)
Patient request	2 (10.5)	0
Unknown	0	2 (4.8)

Percentage of events is calculated based on the total number of reported events. Patients could report multiple events.

### Factors associated with transition to third-line therapy

Chi-squared analyses identified patient sex, steroid use at the start of the data extraction period, steroid use at the end of the data extraction period, choice of second-line treatment, dose optimization of second-line therapy, and treatment choice for first- and second-line therapy as significantly different between patients with CD who remained on second-line treatment compared with those who transitioned to third-line therapy (Table 6). For those with UC, the corresponding factors were steroid use at the end of the data extraction period, choice of second-line treatment and treatment choice for the first and second line.

**Table 6.** Chi-square analysis for correlation between subjects who transitioned to third-line treatment compared with those who remained on second-line therapy.

	Chi-square *P*-value	
Analysis parameter	Crohn’s disease	Ulcerative colitis
Sex	**.013**	.771
Age	.728	.290
Disease duration	.240	.073
EIM at beginning of data extraction period	.540	.917
EIM at end of data extraction period	.342	.927
Use of steroid at beginning of data extraction period	**.001**	.687
Use of steroid at end of data extraction period	**.012**	**.001**
Use of concomitant IMM at beginning of data extraction period	.168	.362
Use of concomitant IMM at end of data extraction period	.462	.985
Use of 5-ASA at beginning of data extraction period	.132	.642
Use of 5-ASA at end of data extraction period	.691	.914
First-line treatment choice	.342	.702
Second-line treatment choice	**.020**	**.001**
First/second line combination	**.001**	**.005**
Dose optimization in first line of therapy	.652	.777
Dose optimization in second line of therapy	.163	**<.001**
Physician years of practise	.450	.250
Physician type of practise	.969	.619

A relationship between the number of treatments taken (2 vs. 3) and the associated analysis parameter was defined as *P* < .05 (bolded).

Abbreviations: 5-ASA, 5-aminosalicylates; EIM, extra-intestinal manifestation; IMM, immunomodulators (methotrexate and azathioprine).

Multivariable regression analysis identified female and steroid use at the beginning of the data extraction period as being significantly associated with transitioning to third-line therapy for patients with CD; for those with UC, significant associations with the need for third-line treatment were identified for the use of ustekinumab (vs vedolizumab) as second-line therapy, and the use of steroids at the end of the data extraction period (Table 7).

**Table 7.** Logistic regression model outcomes to identify parameters associated with the transition to third-line treatment compared with remaining on second-line therapy.

Analysis parameter	Comparisons	Odds ratio (95% CI)
*Crohn ‘s disease*		
First/second line combination	Adalimumab/ustekinumab	0.43 (0.15, 1.18)
(reference: vedolizumab/ustekinumab)	Infliximab/adalimumab	2.25 (0.81, 6.27)
	Infliximab/ustekinumab	0.51 (0.18, 1.46)
	Infliximab/vedolizumab	1.57 (0.55, 4.46)
	Vedolizumab/adalimumab	0.74 (0.22, 2.50)
Sex (reference: male)	Female	2.14 (1.21, 3.78)
Use of steroid at beginning of data extraction period (reference: yes)	No	0.40 (0.23, 0.70)
*Ulcerative colitis*		
Second-line treatment (reference:	Adalimumab	1.12 (0.56, 2.22)
vedolizumab)	Infliximab	1.39 (0.80, 2.42)
	Tofacitinib	0.58 (0.19, 1.76)
	Ustekinumab	0.16 (0.06, 0.44)
Use of steroid at end of data extraction period (reference: yes)	No	0.12 (0.03, 0.41)

The logistic regression models the probability of having a third-line treatment if every other parameter is the same. An odds ratio greater than 1 indicates a greater probability of having a third-line treatment.

## Discussion

To our knowledge, the RECORDED study provides the first data on real-world sequencing and optimization patterns of advanced treatments for moderate-to-severe IBD patients in Canada who received at least 2 advanced therapies. Lack of efficacy and adverse events were the most common reasons for discontinuation of first-line therapy, which occurred at a median (95%CI) duration of 12.3 (10.9, 13.6) months for CD and 9.2 (8.2, 10.8) months for UC. Most patients (78.5% for CD; 65.1% for UC) remained on their second-line therapy for the remainder of the data capture period. Second-line therapy was typically from a different class compared with first-line.

Results from this study should be interpreted in the context of patient cohort selection: all patients included in this study had failed at least their first-line advanced therapy. Therefore, the results from RECORDED should not be used to infer relative efficacy amongst biological treatment naïve patients. TNF antagonists were the most commonly used biologic for first-line therapy for both CD and UC, followed by ustekinumab for CD and vedolizumab for UC, which may reflect prescribers’ familiarity with these therapies which had been available for a longer time. As we did not track patients who remained on first-line therapy it is difficult to assess the validity of this interpretation.

The median (95% CI) time to permanent discontinuation of first-line therapy—12.3 (10.9, 13.6) months for patients with CD and 9.2 (8.2, 10.8) months for those with UC—was comparable for all advanced therapies included in the analysis. Other published real-world evidence (which in contrast to these data, included patients who stayed on their first-line drug) has reported a longer persistence on therapy in patients with UC^16–19^ and CD.^20–22^ These reports are more comparable with the results observed for discontinuation of second-line therapy, given the selection criteria used in this study. The discontinuation rates for second- and third-line treatment reflect both primary and secondary non-responders: in our survival curves, we demonstrate that discontinuation rates of second-line therapy after 12 months are relatively low.

Dose optimization before discontinuation was low, especially considering that loss of efficacy was cited as the reason for discontinuation in approximately 62% and 75% of events in the CD and UC cohorts, respectively. This may reflect the influence of adverse events and other non-loss of response reasons for first-line treatment discontinuation (which would render any further attempts at dose optimization moot). The benefit of dose optimization with TNF antagonists is relatively well established, particularly given the high rates of potential immunogenicity, and association between therapeutic drug concentrations with loss of response over time.^23–25^ However, the same concepts are not necessarily applicable to therapy with vedolizumab, ustekinumab, or future monoclonal therapies, including those targeting IL23p19. For example, the recent ENTERPRET trial demonstrated that dose optimization of vedolizumab was not more effective than standard dosing of patients with early nonresponse and high drug clearance.^26^ In the STARDUST trial, early treatment optimization of ustekinumab in a “treat-to-target” strategy was not more effective than standard care for achieving endoscopic remission.^27^ In the randomized, double-blinded POWER trial, patients with loss of response to ustekinumab did not have improved outcomes with intravenous reinduction during maintenance therapy.^28^ Collectively, these studies highlight that although dose optimization is frequently used as a therapeutic strategy in real-world practice, there is limited controlled evidence to suggest that this is an effective treatment strategy, at least on the population level. Furthermore, vedolizumab and ustekinumab do not have dose escalation as part of their indicated use.^29,30^

Persistence to second-line therapy was higher in the CD cohort. There was no difference in the proportion of patients on combination immunosuppressive therapy with first-line, second-line, or third-line advanced therapies. Despite this, the reasons for treatment change were remarkably consistent across lines of therapy: lack of efficacy and adverse events were the most common reasons for treatment change in similar proportions of cases for first and second-line use. Females with CD were more likely to transition from second to third-line advanced therapy than males, and that steroid use at the initiation of first-line advanced therapy (the start of the data extraction period) was also a predictor of such a transition. For patients with UC, it is unsurprising that steroid use at the end of the data extraction period was a predictor of the need to transition to third-line therapy. Given that steroids are typically used as rescue therapy or for patients perceived to have more severe diseases, it would seem logical to predict that such patients were more likely to be treatment-resistant and unlikely to achieve disease control with their third-line therapy.

Treatment change patterns align with Canadian guidelines, although for patients with CD, ustekinumab would appear to be the treatment of choice after TNF antagonist failure, while vedolizumab appeared to be the most preferred second-line option with UC. Treatment availability at the time of the treatment switch may potentially explain this in UC, given that ustekinumab was not approved for use in UC in Canada until January 2020.

Interpretation of these results should consider that real-world, retrospective studies are by nature observational, uncontrolled, and nonrandomized, and missing data limits the interpretation of some endpoints. While efforts were made to ensure the 22 study sites represented the spectrum of IBD treatment centres across Canada, they may not have captured the full range of treatment patterns. Limiting eligibility to patients who had received treatment of moderate-to-severe IBD with more than 1 advanced therapy (ie, must have failed their first line advanced therapy) enables meaningful real-world analysis of time to and patterns of treatment change, but obviates any comparison with reports of overall real-world time on therapy and limits comparisons between the results presented here for first- and second-line therapies. It may also have introduced bias by selecting patients with longer disease duration. Further, no a priori controls were implemented regarding the use of concomitant therapies including conventional therapies, which may have introduced bias. More information on concomitant use of steroids, for example, may have provided valuable insights as to the timing, duration and dose used which in turn would reduce the potential for bias. Last, the data extraction period was chosen to ensure the treatment patterns for advanced therapies were relevant to current clinical practice; however, the recent expansion of the treatment armamentarium for IBD may limit the applicability of these data. Excluding patients with CD who had prior surgery may also have resulted in selection bias. The lack of therapeutic drug monitoring results and specific capture of ADAs also limit data interpretability, especially for the TNF antagonists.

## Conclusion

The RECORDED study has provided insights into the real-world sequencing and optimization patterns of advanced treatments in patients with moderate-to-severely active IBD in Canada. Levels of dose optimization for first-line therapies prior to discontinuation were lower than anticipated, and second-line therapy was typically from a different class compared with the first-line treatment choice. Further studies should seek to identify the physician and patient-related factors associated with dose optimization of advanced therapies, when appropriate. Finally, given the expanded options of treatment available in 2024 finding the optimal sequence of therapies should be a research priority in IBD.

## Supplementary material

Supplementary material is available at *Journal of the Canadian Association of Gastroenterology* online.

gwae040_suppl_Supplementary_Materials

## Data Availability

Data are available on request.
