# Supplementary material for: Treatment patterns for advanced therapies in Canadians with moderate-to-severe inflammatory bowel disease: a retrospective cohort analysis
Source: J Can Assoc Gastroenterol. 2024 Oct 29;8(1):21–30. doi: 10.1093/jcag/gwae040 (PMC11788506; doi:10.1093/jcag/gwae040)

# Targownik et al (2024). Sequencing of Biologics in Inflammatory Bowel Disease in Canada

## Supplementary information

Table S1. Health Canada notice of compliance dates for biologics and tofacitinib for ulcerative colitis and Crohn’s disease in adults.

| **Generic name** | **NOC date** | **Indication** |
| --- | --- | --- |
| *Ulcerative Colitis* | | |
| Adalimumab | 21^st^ November 2013 | Treatment of adult patients with moderately to severely active UC who have had an inadequate response to conventional therapy including corticosteroids and/or azathioprine or 6-mercaptopurine or who are intolerant to such therapies. The efficacy of Humira in patients who have lost response to or were intolerant to TNF blockers has not been established. |
| Infliximab | 10^th^ March 2006 | Reduction of signs and symptoms, induction and maintenance of clinical remission and mucosal healing, and reduction or elimination of corticosteroid use in adult patients with moderately to severely active UC who have had an inadequate response to conventional therapy (i.e., ASA and/or corticosteroid and/or an immunosuppressant). |
| Ustekinumab | 23^rd^ January 2020 | Treatment of adult patients with moderately to severely active UC who have had an inadequate response with, lost response to, or were intolerant to either conventional therapy or a biologic or have medical contraindications to such therapies. |
| Vedolizumab | 29^th^ January 2015 | Treatment of adult patients with moderately to severely active UC who have had an inadequate response, loss of response to, or were intolerant to either conventional therapy or infliximab, a TNFα antagonist. |
| Tofacitinib | 4^th^ October 2018 | Treatment of adult patients with moderately to severely active UC with an inadequate response, loss of response or intolerance to either conventional UC therapy or a TNFα inhibitor. |
| *Crohn’s Disease* | | |
| Adalimumab | 5^th^ July 2007 | Reducing signs and symptoms and inducing and maintaining clinical remission in adult patients with moderately to severely active Crohn’s disease who have had an inadequate response to conventional therapy, including corticosteroid and/or immunosuppressants. Humira is indicated for reducing signs and symptoms and inducing clinical remission in these patients if they have also lost response to or are intolerant to infliximab. |
| Infliximab | 6^th^ June 2001 | Reduction of signs and symptoms, induction and maintenance of clinical remission and mucosal healing and reduction of corticosteroid use in adult patients with moderately to severely active Crohn’s disease who have had an inadequate response to a corticosteroid and/or aminosalicylate. Remicade® can be used alone or in combination with conventional therapy.  Treatment of fistulising Crohn’s disease, in adult patients who have not responded despite a full and adequate course of therapy with conventional treatment. |
| Ustekinumab | 14^th^ December 2016 | Treatment of adult patients with moderately to severely active Crohn's disease, who have had an inadequate response, loss of response to, or were intolerant to either immunomodulators or one or more TNFα antagonists, or have had an inadequate response, intolerance or demonstrated dependence on corticosteroids. |
| Vedolizumab | 22^nd^ March 2016 | Treatment of adult patients with moderately to severely active Crohn’s disease who have had an inadequate response with, lost response to or were intolerant to a TNFα antagonist or immunomodulators, or had inadequate response, intolerance or demonstrated dependence on corticosteroids. |

NOC, Notice of Compliance; TNF, tumor necrosis factor; UC, ulcerative colitis. Data extracted from Health Canada (<https://health-products.canada.ca/noc-ac/?lang=eng>).

Table S2. Investigators and sites for the RECORDED study.

| **Investigator** | **Treatment centre** | **Centre type** | **Patients enrolled CD/UC/TOTAL** |
| --- | --- | --- | --- |
| Vipul Jairath | London Health Sciences Center- University Hospital, London ON | Academic | 25/21/46 |
| Louis-Charles Rioux | CIUSSS de l'est de l'Ile-de-Montreal - Hopital Maisonneuve-Rosemont, Montreal QC | Community | 10/14/24 |
| Kaleb Marr | Six08 Gastroenterology, Lethbridge AB | Community | 21/18/39 |
| Bruce Musgrave | Bruce Musgrave Medical Services Inc., Kentville NS | Community | 14/10/24 |
| Allen Lim | Gastroenterology and Internal Medicine Research Institution, Edmonton AB | Community | 8/10/18 |
| Marie-Diane Stewart | Gastro-Entérologie des Laurentides, St-Jerome QC | Rural | 9/7/16 |
| Robert Berger | R&M Berger Medical Inc., Moncton NB | Community | 21/16/37 |
| Robert Bailey | GI Research & Associates, Edmonton AB | Community | 14/8/22 |
| Smita Halder | Hamilton Health Sciences - McMaster University Medical Centre, Hamilton ON | Community | 13/33/46 |
| Christopher Ma | University of Calgary, Calgary AB | Academic, Community | 1/1/2 |
| Ian Bookman | Kensington Screening Clinic, Toronto ON | Academic | 10/11/21 |
| Mark MacMillan | Dr. Everett Chalmers Reg Hosp., Fredericton NB | Academic | 21/18/39 |
| Scott Shulman | Scott Shulman Medicine Professional Corporation, North Bay ON | Academic | 21/28/49 |
| Daniel Green | Taunton surgical centre, Oshawa ON | Academic | 18/24/42 |
| Kenneth Atkinson | Fraser Clinical Trials Inc, New Westminster BC | Community | 16/14/30 |
| Jesse Siffledeen | Dr. J Siffledeen Profess Pract, Edmonton AB | Academic, Community, Rural | 3/8/11 |
| Waqqas Afif | Royal Victoria Hospital / McGill University Health Centre, Montreal QC | Community | 29/19/48 |
| Sundeep Singh | OCT Research ULC, Kelowna BC | Academic | 2/9/11 |
| David Ford | David Ford Medicine Professional Corporation, Woodbridge ON | Academic | 28/33/61 |
| John Igoe | The Moncton Hospital, Moncton NB | Academic | 12/17/29 |
| Edmond-Jean Bernard | CHUM - Centre hospitalier de l'Universite de Montréal, Montreal QC | Community | 2/2/4 |
| Laura Targownik | Mount Sinai Hospital, Toronto ON | Academic | 32/23/55 |

CD, Crohn’s disease; FAS, full analysis set; TOT, total; UC, ulcerative colitis. Number of patients enrolled represents numbers included in the FAS.

Table S3.Time from permanent discontinuation of first line and initiation of second line advanced therapy.

|  | **Adalimumab (N=230)** | **Infliximab  (N=256)** | **Ustekinumab (N=43)** | **Vedolizumab (N=145)** | **Overall (N=674)** |
| --- | --- | --- | --- | --- | --- |
| *Crohn’s disease* | | | | | |
| n | 83 | 82 | 32 | 39 | 236 |
| Median (days) | 21.0 | 25.0 | 17.0 | 33.0 | 23.0 |
| *Ulcerative colitis* | | | | | |
| n | 69 | 98 | 1 | 72 | 240 |
| Median (days) | 16.0 | 25.5 | 32.0 | 16.0 | 19.0 |

Figure S1. Time to permanent discontinuation of first line treatment – Crohn’s disease.


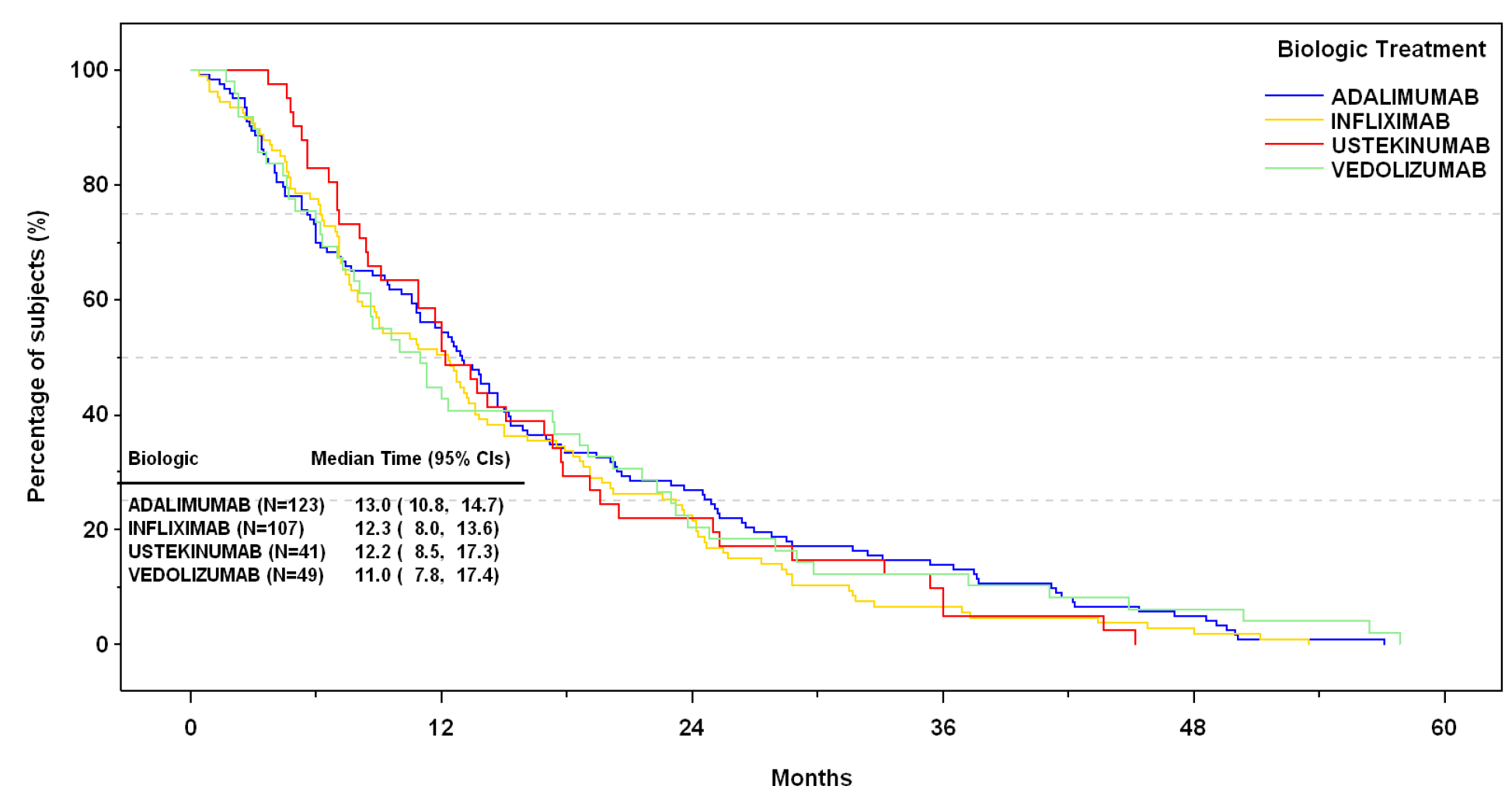


Figure S2. Time to permanent discontinuation of first line-treatment – ulcerative colitis.


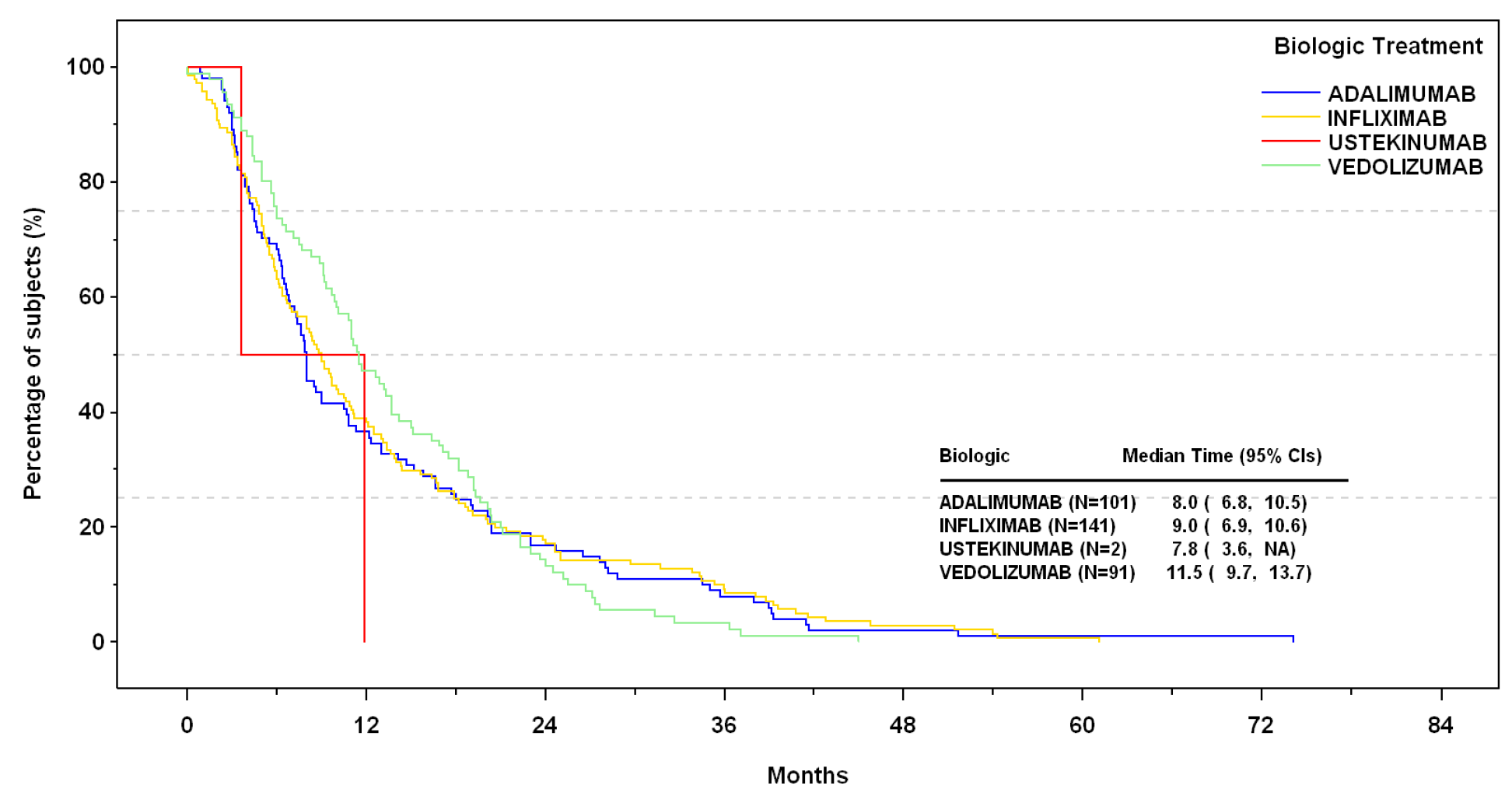


Figure S3. Time to first treatment optimization of first line treatment – Crohn’s disease.


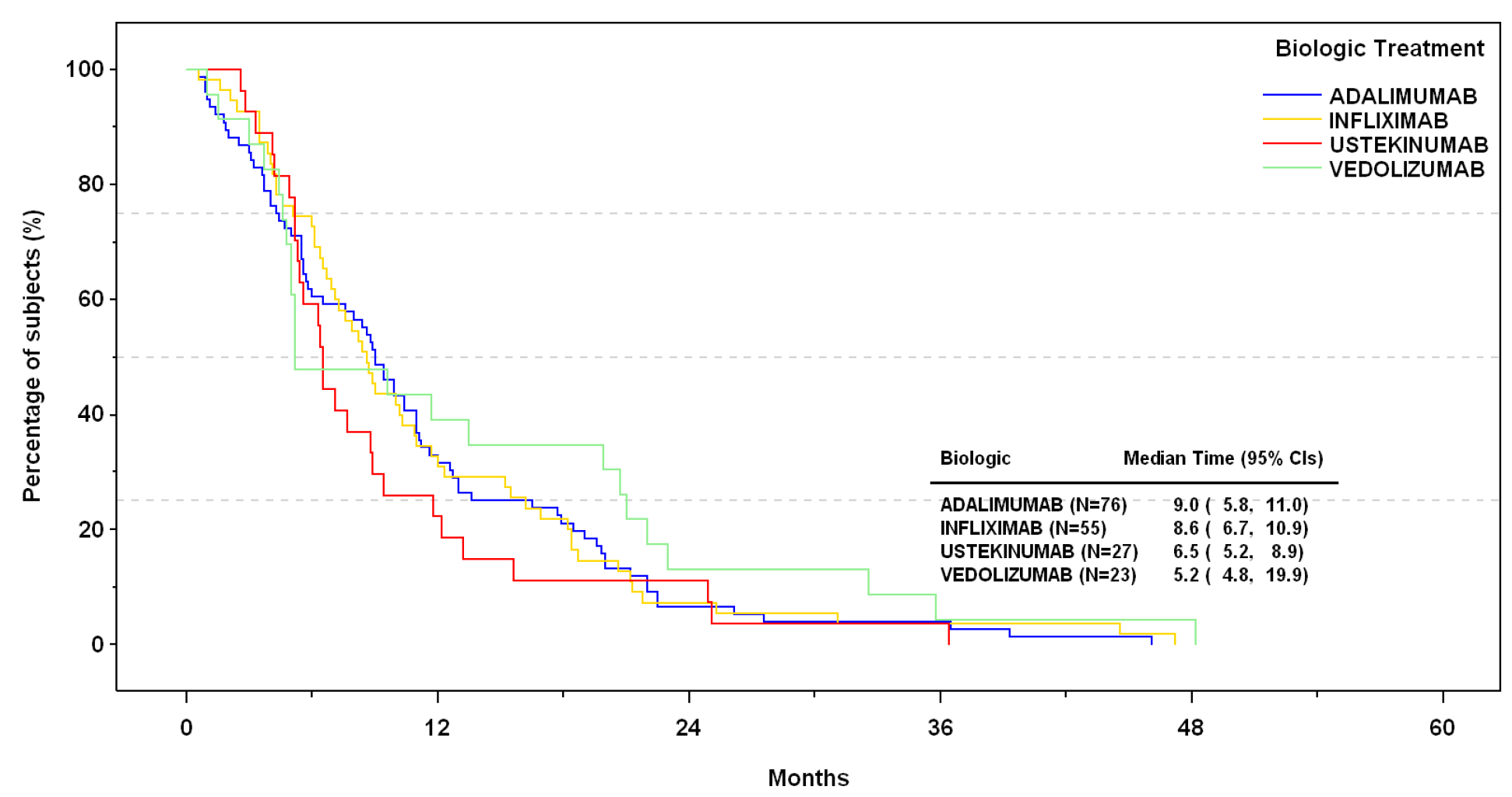


Figure S4. Time to first treatment optimization of first line treatment – ulcerative colitis.


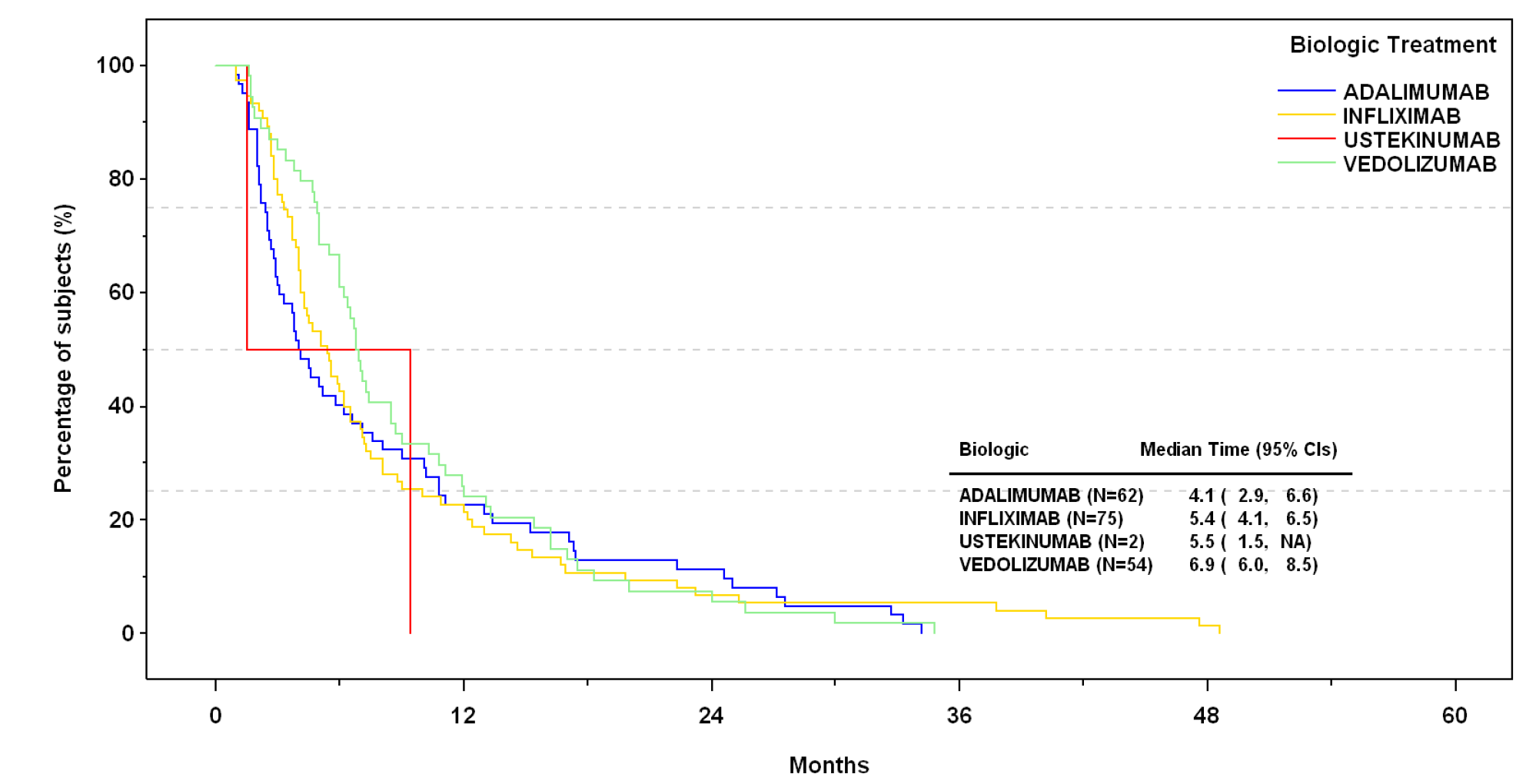

Supplement: gwae040_suppl_Supplementary_Materials [file gwae040_suppl_supplementary_materials.zip › RECORDED manuscript supplementary information V06 06AUG2024.docx]
